# Optimal time between decompressive craniectomy and cranioplasty to reduce the risk of complications: A retrospective study

**DOI:** 10.1007/s10143-025-03993-1

**Published:** 2026-01-02

**Authors:** Federica Stretti, Richard Parvin, Mateo Tomas Fariña Nuñez, Ignazio De Trizio, Massimo Barbagallo, Laura Tatsch, Johannes Sarnthein, Giuseppe Esposito, Clelia Di Serio, Alberto Pagnamenta, Giovanna Brandi

**Affiliations:** 1 Institute for Intensive Care Medicine, University Hospital Zurich, Zurich, Switzerland; 2 Department of Neurosurgery, University Hospital Zurich, Zurich, Switzerland; 3 Clinical Neuroscience Center, University Hospital Zurich, Zurich, Switzerland; 4 University Centre for Statistics in the Biomedical Sciences, Vita Salute San Raffaele University, Milan, Italy; 5 Clinical Trial Unit, Ente Ospedaliero Cantonale, Lugano, Switzerland; 6 Department of Intensive Care, Ente Ospedaliero Cantonale, Lugano, Switzerland; 7 Division of Pneumology, University of Geneva, Geneva, Switzerland; 8 Department of Orthopaedic Surgery, Neurosurgery and Spine Surgery, Schulthess Clinic, Zurich, Switzerland; 9 Neuro- and Spine Center, Lucerne, Switzerland

**Keywords:** Cranioplasty, Decompressive hemicraniectomy, Intracranial complications, Timing

## Abstract

Cranioplasty (CP) is performed after decompressive craniectomy (DC), with complication rates of 15-40%. Risk factors and ideal timing for CP are unclear. This study investigates risk factors for complications after CP and the best time to perform a CP. This retrospective single-center study includes patients admitted to the neurosurgical department of the University Hospital Zurich between 01.2013 and 05.2023, who underwent a CP following a supratentorial DC. Demographics, comorbidities, indication for the DC, material used, complications, time between DC, CP and onset of complication were collected. The investigated complications are infections, bleeding, bone flap resorption or others requiring surgery. A multivariable logistic regression was performed to identify risk factors for the occurrence of complications. A Classification and Regression Tree (CART) was performed to identify the best time for CP. The result was tested with a Cox regression and a ROC analysis. Overall, 141 patients were included, of whom 39% (n=55) developed a complication. No difference in sex, age, comorbidities, DC-indication, material used was found, except for a trend for CP-time (shorter time for patients with vs without complication, 84±44 vs 102±70 days, p=0.065). In the CART analysis, day 122.5 was the best time cut-off for reducing complications (sensitivity 87%). In our case-series, we could not identify risk factors for complications after CP, except possibly for CP-timing. A longer time between DC and CP seems to be associated with less complications, with day 122.5 as the cut-off time.

**Supplementary information:**

The online version contains supplementary material available at 10.1007/s10143-025-03993-1.

## Introduction

Decompressive craniectomy (DC) is a life-saving neurosurgical procedure in the management of refractory intra-cranial hypertension secondary to acute brain injury (ABI), as ischemic stroke, intracranial bleeding or traumatic brain injury (TBI) [1]. After the acute phase, when the cerebral edema has subsided, cranial reconstruction through cranioplasty (CP) is required [2–6] in order to restore skull integrity for cerebral protection, to reestablish a more physiological cerebral blood flow and cerebral spinal fluid (CSF) dynamics, and also for cosmetic purposes. Despite the relative simplicity of the procedure, CP is associated with high complication rates of 15–40% [7, 8] compared to other elective neurosurgical procedures, and the occurrence of complications can affect the functional outcome [9]. DC per se exposes patients to three types of major complications: hemorrhagic, infectious/inflammatory and disturbances of the CSF compartment [10]. Complications associated with CP are similar, with additional problems related to the bone flap, as bone flap resorption.

The best timing for CP is unclear and varies from center to center. Some studies support early CP within 3 months after DC [11], whereas other studies encourage to wait longer, reporting higher risk of surgical complications in case of surgery within 3 months [12, 13].

The present retrospective cohort study aims to identify risk factors for the development of complications post-CP and to locate the best cut-off time to perform a CP to reduce the occurrence of complications.

## Material & methods

This study was performed in line with the principles of the Declaration of Helsinki. Approval was granted by the Cantonal Ethics Committee Zurich (KEK PB-2017-00093). Informed consent was obtained from all individual participants included in the study or their next of kin of case of incapacity. STROBE guidelines were used to draft the manuscript. Data may be made available upon reasonable request. Clinical trial number: not applicable.

All patients who underwent CP after supratentorial DC for ABI at the Neurosurgery department of the University Hospital Zurich between January 2013 and May 2023 were considered eligible for inclusion in this retrospective study. Inclusion criteria were: (1) adults (aged > 18 years), (2) surgery for CP after supratentorial DC due to ABI performed at the University Hospital Zurich. Exclusion criteria were (1) patient’s written or documented oral refusal to have their data analyzed for research projects; (2) surgery for DC and/or CP performed in another hospital.

Data on demographics, imaging, laboratory values, operative reports, and hospital discharge letters were obtained from the patient registry of the Neurosurgery department [14] and from the hospital electronic health records (KISIM-TM, Cistec^®^ Zurich, Switzerland). Collected demographic data were: sex, age, presence of comorbidities at hospital admission, based on the Charlson Comorbidity Index (CCI) [15], use of anticoagulation/anti-platelet drugs.

The indication for DC included: hemorrhagic stroke, ischemic stroke, traumatic brain injury (TBI), central nervous system (CNS) infections and others (e.g. tumors). Skull reconstruction with CP included autologous bone graft or alloplasts in polyetheretherketone (PEEK plastic).

The following complications after CP were assessed: central nervous system infection -confirmed by positive CSF or brain tissue culture-, relevant cerebral bleeding -defined by the need of re-surgery- or bone resorption with need of re-surgery (for patients who underwent autologous bone graft only) and others (e.g. oedema, CSF fistula, leakage, dehiscence) with need of re-surgery. Time between DC and CP as well as time between CP and onset of complication was collected and expressed in days.

### Statistical analysis

Descriptive statistics are reported as counts/percentages, mean ± standard deviation (SD), or as median including the corresponding 25th and 75th percentiles, as appropriate. Comparisons between patients without intracranial complication and patients with complication were performed with Pearson’s χ^2^ or Fisher’s exact test, Student’s *t*-tests or Mann–Whitney U, as appropriate. To identify potential predictors of complications after CP, we performed a multivariable logistic regression analysis. In the absence of a pre-specified hypothesis concerning the effects of the time frame between DC and CP for the occurrence of complications, we built a Classification and Regression Tree (CART) to identify the best cutoff entering the following variables as candidate predictors of postoperative complications: time interval between DC and CP, gender, age. The CART approach was chosen because of the limited sample size and the exploratory nature of the study, aimed at identifying an interpretable clinical threshold. The obtained cutoff of time was further tested in a multivariable Cox proportional hazards regression analysis by splitting the whole cohort into two according to this cutoff value. A Receiver Operating Characteristic (ROC) analysis permitted to quantify sensitivity and specificity at optimal cut-off, to provide an indication of its discriminative ability.

All statistical analyses were performed two-sided, and p-value < 0.05 was considered statistically significant. Statistical analyses were performed using Stata software (version 17.0; StataCorp LLC, College Station, TX, USA). Additional graphical and ROC analyses were performed in R (version 4.3.2; R Foundation for Statistical Computing, Vienna, Austria). No handling of missing data was necessary (dataset complete). As for the assumption of proportional hazards, this was verified graphically and with Schoenfeld’s test.

## Results

Overall, 141 patients fulfilled the inclusion criteria of the study, of them 76 (54%) were male. Mean age was 49 ± 14 years. Reasons for DC were hemorrhagic stroke (*n* = 49, 35%), ischemic stroke (*n* = 33, 23%), TBI (*n* = 49, 35%), CNS-infections (*n* = 5, 4%), or others (e.g. brain tumors) (*n* = 5, 4%) (Table 1).

**Table 1:** Patients’ demographics

	Overall	Patients with complications	Patients without complications	*p* value
n. patients (%)	141	55 (39%)	86 (61%)	
Age (years, mean ± SD)	49 ± 14	47 ± 15	50 ± 13	0.267
Sex (n. male pts (%))	76 (54%)	30 (55%)	46 (54%)	0.902
INDICATION FOR DC (n. pts %)				
Hemorrhagic stroke	49 (35%)	15(29%)	33 (38%)	0.247
Ischemic stroke	33(23%)	12 (22%)	21 (24%)	
TBI	49 (35%)	25 (45%)	24 (28%)	
Infection	5(4%)	1 (2%)	4 (5%)	
Others	5 (4%)	1 (2%)	4 (5%)	
CRANIOPLASTY (n. pts %)				
Autologous bone	103 (73%)	43 (78%)	60 (70%)	0.274
PEEK plastic	38 (27%)	12 (22%)	26 (30%)	
Charlson Comorbidity Index (median [IQR])	1 [0–3]	1 [0–3]	1 [1–3]	0.517
Time between DC and CP (days, mean ± SD)	95 ± 62	84 ± 44	102 ± 70	0.065

Table 1. Patients’ demographics. Comparisons between groups (patients with post CP complications versus patients without complications). *DC* decompressive craniectomy, *TBI* traumatic brain injury, *PEEK* polyetheretherketone, *CP* cranioplasty. Data are expressed as mean ± standard deviation (SD) or median and interquartile range (IQR), as appropriate

Approximately a third of the patients developed a complication after CP (*n* = 55; 39% Table 2). Of these, 14 (25.5%) developed a relevant cerebral bleeding, 14 (25.5%) developed a CNS infection, 23 (42% of patients who developed complications, 22% of the patients (*n* = 103) who received autologous bone graft) bone resorption and 4 other complications (7%). Patients who developed a post-CP complication did not differ for age (*p* = 0.267), CCI (*p* = 0.517), indication for the DC (*p* = 0.247), use of autologous bone vs. allograft (*p* = 0.274) from patients who did not have a complication (Table 1). Patients with complications had a shorter time between DC and CP than patients without reaching statistical significance (mean 84 ± 44 days versus 102 ± 70, *p* = 0.065) (Table 1).

**Table 2:** Complications and their timing

	*N*. of patients *n* (%)	Timing (days) median [IQR]
Overall	55	320[13–636]
Infection	14 (25.5%)	49 [22–114]
Rebleeding	14 (25.5%)	5 [3–10]
Bone resorption	23 (42%)	636 [475–782]
Others	4 (7%)	94 [66–268]

Table 2. Complications and their timing. Timing between the cranioplasty and the occurrence of the complication. IQR interquartile range

Several multivariable logistic regression models were also performed considering among others age, sex, comorbidities based on the CCI, use of anticoagulation/antiplatelet drugs, indication for the DC without significant results (data in Supplementary material). The time interval between DC and CP showed a protective signal against the occurrence of complication without a statistical significance (OR 0.99; 95%-CI: 0.99–1.00.99.00).

**Fig. 1:**
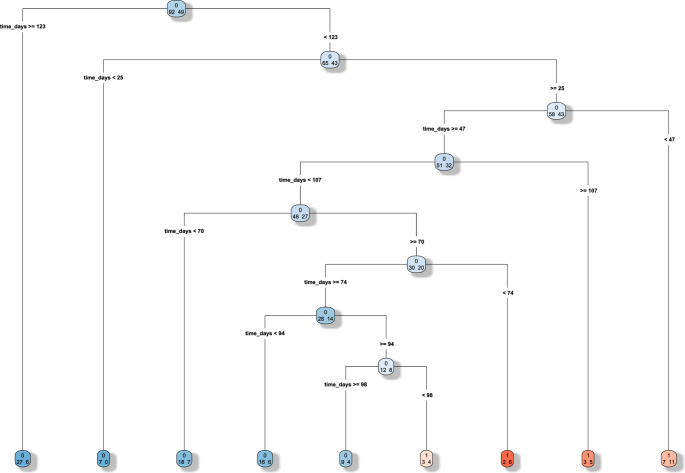

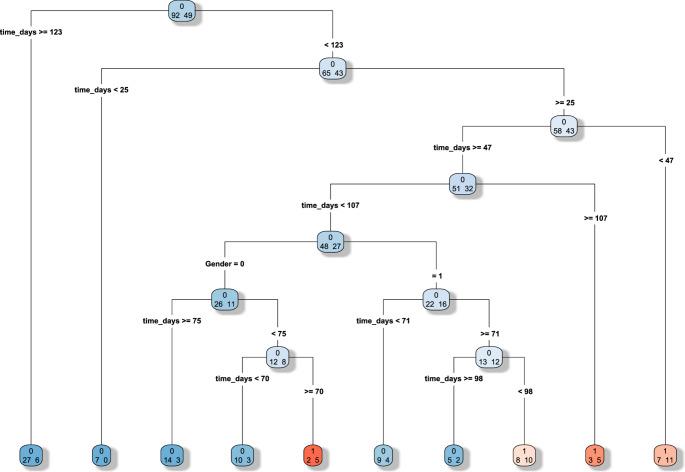

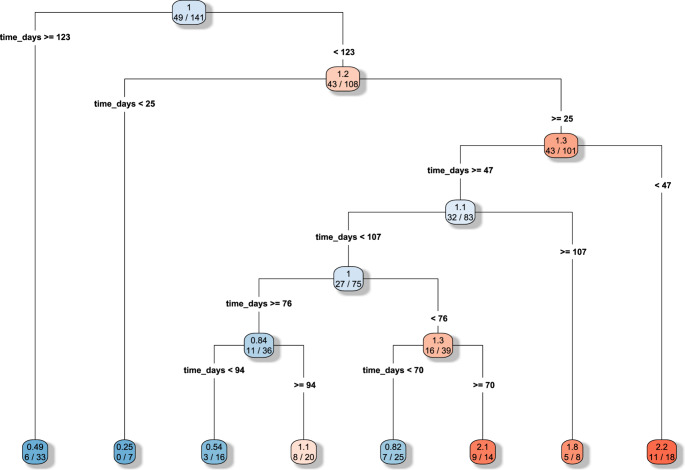
CART analysis. The time cut off at 122.5 days for the occurrence of complications after cranioplasty presents a good sensitivity of 87% and specificity of 30%. Gender 0=female, 1= male

**Fig. 2:**
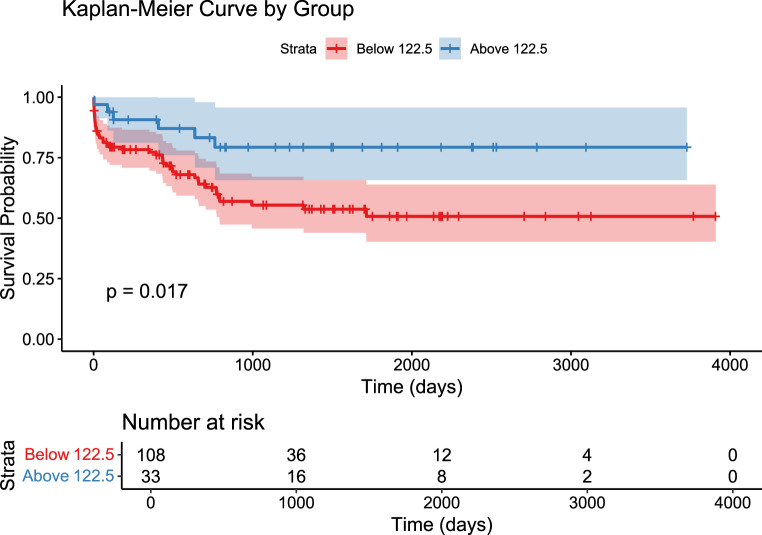
Kaplan Meier curve with log rank test. The study population was split in 2 subpopulations to be compared according to the previously identified cutoff of 122.5 days for the occurrence of complications following cranioplasty. The red curve represents patients who received the CP earlier than day 122.5 post-CP. The blue curve represents patients who received the CP after day 122.5 post-CP

The CART analysis (Fig. 1) identified 122.5 days as the cutoff for the occurrence of complications. The algorithm, based on the Gini impurity index, automatically selected time as the dominant discriminating variable, identifying a main split at 122.5 days, while neither age nor gender contributed meaningfully to the partitioning. The result was further confirmed with the time to event analysis. A ROC analysis found at optimal cut off a sensitivity of 87% and specificity of 30%. In Fig. 2 we present the Time to event analysis after splitting the study population based on the previously identified cut off between DC and CP of 122.5 days, to obtain two subpopulations to compare.

## Discussion

Optimal timing of CP after hemispheric DC is still a matter of debate. In the present study we investigated possible risk factors for complications in patients who underwent a CP.

As a main result, we could not find risk factors associated with the likelihood of developing complications, except for a trend between a longer time-interval between DC and CP and less postoperative complications. Further analysis identified 122.5 days as the temporal cut-off for the CP to reduce the occurrence of complications. Our finding suggests that in the study population waiting more than four months for the CP could have a positive impact -in terms of less complications- on the patients’ clinical course, provided that there are no clinical conditions preventing the delay in restoring skull integrity, as sinking flap or need to reestablish the CSF dynamic. However, an external validation is needed to confirm the finding.

In our population, we found an overall complication rate of 39%, in line with previous data from the literature [16]. Despite the relative simplicity and brevity of the procedure, cranioplasty is a high-risk procedure needing a reoperation and/or a long duration of antibiotic therapy in about a third of the patients. It is then essential to understand the possible modifiable risk factors to ensure a reduced morbidity and hasten the recovery, especially due to the growing number of these procedures in the last years [17].

A previous systematic review analyzed possible modifiable factors to reduce the incidence of infections post CP. Only 3 studies included investigated the timing of surgery and the review failed to find any statistically significant association with infection when comparing “early” (< 3 months) to “late” (>3 months) CP [18]. However, the small sample size of the population was the main limitation of the analysis. Similarly, a more recent systematic review showed the same rate of infections, reoperations, intracranial hemorrhage, extra-axial fluid collections, seizures, and bone resorption with early vs. late cranioplasty, with limitations due to the heterogeneity of the studies included regarding patients’ population, definition of complications, and time cut off for early and late surgery [16]. Moreover, studies published in more recent times showed an increased rate of complications with early surgery [12, 19], with possibly the only exception of CSF disturbances and bone resorption in trauma patients [20, 21], that has brought to a trend to earlier reimplantation in trauma patients [17].

As an example, a recent retrospective single center study found an association with an increased risk of infections and CP in the “ultra-early” period (< 15 days). The authors suggest to wait to perform a CP between 15 and 90 days after initial craniectomy to minimize the risk of infection, seizure, and bone resorption; while waiting more than 90 days may be associated with a lower risk of hydrocephalus but with an increased risk of seizure [7].

We focused the analysis only on few complications post CP, mainly new CNS infections, relevant cerebral bleeding with need of re-surgery, and bone resorption -for the patients who received autologous bone graft. These variables are clinically relevant (need for surgery with higher costs and burden for the patient with longer hospitalization) and well defined. We think that these complications are closely related to the CP and less to the acute brain injury per se -the reason for the DC- as seizures, for example, could be. These complications have been less investigated so far, as also underlined in a recent review [16].

To our knowledge this is the first study to investigate an optimal time point to reduce the incidence of postoperative complications after CP. In previous studies data were dichotomized according to various definitions of early vs. late CP (15–120 days [16]). In an older study [13], although not significant, there was a reduced incidence of complication after 20 weeks. While it is of paramount importance to optimize the surgical procedure reducing the number of complications and avoiding prolonging the hospitalization, it is also fundamental to avoid an unnecessary delay. In a qualitative study investigating patients awaiting a CP after DC, this waiting period was described as a limbo and patients struggled to find new routines [22].

In our study we included a heterogeneous population of patients with brain injury with an even distribution between trauma, hemorrhagic and ischemic injury. While it has been suggested that early CP could be better for trauma in terms of reduced complications such as sinking flap and fluid collection [16], we did not find a similar trend in our patients` population. It would be of great interest to better investigate the association between CP timing and DC indication.

Due to the retrospective nature, our study design bears limitations. Firstly, this is a retrospective single center study. Therefore, the generalizability of the results may be limited because we cannot exclude detection and referral biases. Secondly, we limited the analysis to few complications following CP, even if it was a choice to focus on complications less investigated so far and with a major impact on the patient’s clinical course. Thirdly, due to the relatively small sample-size (*n* = 141 patients, 55 complications), there are some statistical limitations. In our multivariable logistic regression model, we could consider only a restricted number of potential risk factors. An analysis of the association between the different complications and the reason for the DC, though of great interest, is not feasible. Exploring the differences in the underlying etiology of acute brain injury, and consequently in the indications for DC, could be valuable for a better understanding of the specific role of the surgical procedure itself, as well as the influence of other factors—both patient-specific (e.g., comorbidities) and disease-specific—on the development of post-CP complications and their timing. The identified 122.5-day cutoff was used for descriptive and hypothesis-generating purposes only. We could not perform an internal validation and we fully acknowledge the potential for optimism when the same data are used for both derivation and evaluation. Our results, particularly the 122.5-day threshold, are exploratory and descriptive, warranting confirmation in larger independent cohorts for external validation with a decision-curve analysis.

The strength of this study, however, lies in the use of a database of consecutive patients who underwent a DC and a CP at our center during a 10-year period with several parameters collected, a good characterization of patients’ comorbidities, very few missing data and the objective definition of complications (need for surgery or positive culture).

## Conclusions

Our data suggest that the longer the interval between DC and CP is, the less likely patients are to develop complications as new CNS infections, cerebral bleeding with need of re-surgery, or bone resorption. In our population, the optimal time cut-off to reduce the incidence of postoperative complications was 122.5 days.

## Supplementary information

Below is the link to the electronic supplementary material.

Supplementary File 1(DOCX 35.4 KB)

## Data Availability

No datasets were generated or analysed during the current study.
