# Supplementary material for: Optimal time between decompressive craniectomy and cranioplasty to reduce the risk of complications: A retrospective study
Source: Neurosurg Rev. 2026 Jan 2;49(1):94. doi: 10.1007/s10143-025-03993-1 (PMC12764505; doi:10.1007/s10143-025-03993-1)
Supplement: Supplementary file 1 — (DOCX 35.4 KB) [file 10143_2025_3993_MOESM1_ESM.docx]

**SUPPLEMENTARY MATERIALS**

**1. Multivariate analysis**

Several models were performed according to the findings of the univariate analysis and their clinical relevance. The time interval between decompressive craniectomy and cranioplasty showed a trend toward an association with the occurrence of complications (the shorter the interval, the higher the likelihood of developing a complication).

------------------------------------------------------------------------------------------

complicationyesno | Odds ratio Std. err. z P>|z| [95% conf. interval]

--------------------------+----------------------------------------------------------------

Gender | 1.057117 .3900156 0.15 0.880 .512954 2.17855

Age_during_reimplantation | .9830267 .0134929 -1.25 0.212 .9569337 1.009831

anticoagulationDicotomic | .8384124 .3691113 -0.40 0.689 .3537659 1.987007

Reimplantation | .6260767 .2713742 -1.08 0.280 .2677149 1.46414

time_days | .9958143 .0037372 -1.12 0.264 .9885164 1.003166

_cons | 3.987154 3.913397 1.41 0.159 .5823816 27.29722

-------------------------------------------------------------------------------------------

---------------------------------------------------------------------------------------------

_t | Haz. ratio Std. err. z P>|z| [95% conf. interval]

----------------------------+----------------------------------------------------------------

time_daysDic | .7706191 .2271748 -0.88 0.377 .4324239 1.373314

Gender | .8186277 .2504966 -0.65 0.513 .4493889 1.49125

Age_during_reimplantation | .9789634 .0117857 -1.77 0.077 .9561342 1.002338

anticoagulationDicotomic | .9203114 .3321549 -0.23 0.818 .4536519 1.867011

Myocardial_infarction | .5857147 .4670642 -0.67 0.502 .1227201 2.79548

Liver_disease | 1.607375 .5678629 1.34 0.179 .804266 3.212437

Diabetes_mellitus | 1.900485 1.259606 0.97 0.333 .5184468 6.966664

Peptic_ulcer_disease | 2.825693 1.523396 1.93 0.054 .9822516 8.128815

CHF | 2.032202 2.238956 0.64 0.520 .2345122 17.61037

COPD | 1.302371 .5974741 0.58 0.565 .5299543 3.200596

peripheral_vascular_disease | 4.727888 4.952331 1.48 0.138 .6068165 36.83639

---------------------------------------------------------------------------------------------

------------------------------------------------------------------------------------------

_t | Haz. ratio Std. err. z P>|z| [95% conf. interval]

--------------------------+----------------------------------------------------------------

time_daysDic | .7282795 .2096419 -1.10 0.271 .414259 1.280337

Gender | .9585445 .2872436 -0.14 0.888 .532765 1.724602

Age_during_reimplantation | .9878821 .0108261 -1.11 0.266 .9668895 1.00933

anticoagulationDicotomic | .8700159 .296569 -0.41 0.683 .4460377 1.697004

Myocardial_infarction | .7333149 .5726595 -0.40 0.691 .1586978 3.388519

Liver_disease | 1.738839 .5256425 1.83 0.067 .9614911 3.144658

-------------------------------------------------------------------------------------------

---------------------------------------------------------------------------------------------

_t | Haz. ratio Std. err. z P>|z| [95% conf. interval]

----------------------------+----------------------------------------------------------------

time_daysDic | .4270336 .179818 -2.02 0.043 .1870823 .9747456

Gender | .7911586 .2447743 -0.76 0.449 .4314323 1.450823

Age_during_reimplantation | .9778948 .0117049 -1.87 0.062 .9552207 1.001107

anticoagulationDicotomic | 1.086047 .39124 0.23 0.819 .5360554 2.200327

Myocardial_infarction | .8079967 .6522963 -0.26 0.792 .1660481 3.931743

Liver_disease | 1.602317 .572671 1.32 0.187 .7952937 3.228267

Diabetes_mellitus | 1.969642 1.297671 1.03 0.304 .5414835 7.16456

Peptic_ulcer_disease | 2.80237 1.478544 1.95 0.051 .9963857 7.881766

CHF | 1.914379 2.076229 0.60 0.549 .2284872 16.03961

COPD | 1.468916 .6810316 0.83 0.407 .592046 3.644504

peripheral_vascular_disease | 6.727418 7.148751 1.79 0.073 .8381792 53.9958

CKD | 1 (omitted)

---------------------------------------------------------------------------------------------

-------------------------------------------------------------------------------------------

_t | Haz. ratio Std. err. z P>|z| [95% conf. interval]

--------------------------+----------------------------------------------------------------

time_daysDic | .4556776 .1872609 -1.91 0.056 .2036368 1.019669

Gender | .9491128 .2847373 -0.17 0.862 .5271743 1.708761

Age_during_reimplantation | .9874664 .0107771 -1.16 0.248 .966568 1.008817

anticoagulationDicotomic | 1.016316 .3531439 0.05 0.963 .5143492 2.008166

Myocardial_infarction | .8624744 .6795977 -0.19 0.851 .1840904 4.040744

Liver_disease | 1.769047 .5338875 1.89 0.059 .9791558 3.196147

-------------------------------------------------------------------------------------------

**2. Analysis according to different brain injury etiology**

*
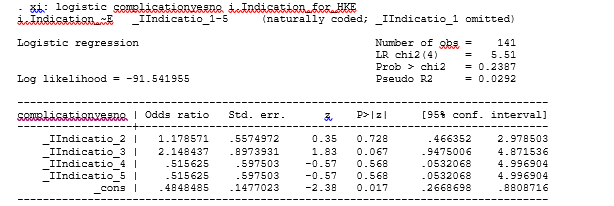
*

Indication 1: Hemorrhagic stroke, 2 ischemic stroke, 3 Traumatic Brain Injury, 4 infection, 5 others.

**3. Complications and demographics in subgroups.** (early vs late, using 122.5 days as cutoff and by material used (autologous bone vs PEEK)).

|  | Autologous | PEEK |
| --- | --- | --- |
| N of patients | 103 | 38 |
| Gender (male, n (%)) | 54 (52%) | 22 (58%) |
| Age (years, median (IQR)) | 53 (43-60) | 46 (33-53) |
| Complication n (%) | 43 (42%) | 12 (32%) |
| Infection | 11 | 3 |
| Bleeding | 7 | 7 |
| Autolytic bone | 23 | 0 |
| Others | 2 | 2 |
| Timing (days) | 636 (154-1438) | 921 (90-1675) |

|  | Early | Late |
| --- | --- | --- |
| N of patients | 108 | 33 |
| Gender (male, n (%)) | 55 (51%) | 21 (64%) |
| Age (years, median (IQR)) | 51(37-59) | 55 (46-61) |
| Complication n (%) | 48 (44%) | 7 (21%) |
| Infection | 13 | 1 |
| Bleeding | 12 | 2 |
| Autolytic bone | 20 | 3 |
| Others | 3 | 1 |
